# Finding local communities in protein networks

**DOI:** 10.1186/1471-2105-10-297

**Published:** 2009-09-18

**Authors:** Konstantin Voevodski, Shang-Hua Teng, Yu Xia

**Affiliations:** 1 Department of Computer Science, Boston University, Boston, MA 02215, USA; 2 Microsoft Research New England, Cambridge, MA 02142, USA; 3 Bioinformatics Graduate Program, Boston University, Boston, MA 02215, USA; 4 Department of Chemistry, Boston University, Boston, MA 02215, USA

## Abstract

**Background:**

Protein-protein interactions (PPIs) play fundamental roles in nearly all biological processes, and provide major insights into the inner workings of cells. A vast amount of PPI data for various organisms is available from BioGRID and other sources. The identification of communities in PPI networks is of great interest because they often reveal previously unknown functional ties between proteins. A large number of global clustering algorithms have been applied to protein networks, where the entire network is partitioned into clusters. Here we take a different approach by looking for local communities in PPI networks.

**Results:**

We develop a tool, named Local Protein Community Finder, which quickly finds a community close to a queried protein in any network available from BioGRID or specified by the user. Our tool uses two new local clustering algorithms Nibble and PageRank-Nibble, which look for a good cluster among the most popular destinations of a short random walk from the queried vertex. The quality of a cluster is determined by proportion of outgoing edges, known as conductance, which is a relative measure particularly useful in undersampled networks. We show that the two local clustering algorithms find communities that not only form excellent clusters, but are also likely to be biologically relevant functional components. We compare the performance of Nibble and PageRank-Nibble to other popular and effective graph partitioning algorithms, and show that they find better clusters in the graph. Moreover, Nibble and PageRank-Nibble find communities that are more functionally coherent.

**Conclusion:**

The Local Protein Community Finder, accessible at http://xialab.bu.edu/resources/lpcf, allows the user to quickly find a high-quality community close to a queried protein in any network available from BioGRID or specified by the user. We show that the communities found by our tool form good clusters and are functionally coherent, making our application useful for biologists who wish to investigate functional modules that a particular protein is a part of.

## Background

Using the link structure of a network to gain insight into the function of its nodes is a ubiquitous technique in biological, social, and computer networks [1-11]. For example, Kleinberg used the link structure of the Internet to give each node a hub and an authority index [9], and Brin and Page utilized the structure of the Web, rather than its content, to rank Web pages [10,11]. Of particular interest is the identification of network communities, also in the context of the Internet [6-8], and social and biological networks [1-5]. Communities are especially relevant in protein-protein interaction (PPI) networks because they often represent protein complexes or other modules with related function.

There are several ways to define a community in a network. One definition is to say that a community is a group of highly interconnected nodes. Finding large cliques in a graph, which are subsets of nodes that are completely connected, is a well-studied problem. It is computationally infeasible for large networks: finding the size of the largest clique in a graph is NP-Complete [12], and approximating it is hard as well [13]. Thus many heuristic methods have been developed, which look for defective cliques (cliques that are missing some edges), or more generally dense components.

In the context of biological networks, Bu et al. use spectral analysis of the adjacency matrix of a graph to find nodes in cliquish components [14]. They apply this technique to yeast PPI networks to identify imperfect cliques, in order to predict the functions of the unknown proteins that they contain. In addition to using spectral approaches, a simpler strategy for finding defective cliques is to first enumerate maximal cliques using an exact (but time consuming) solution, and then combine them if they have significant overlap [15].

A different way to characterize a community is to look at it as a cluster: a group of nodes that are highly interconnected among themselves, but sparsely connected to the rest of the network. The quality of a cluster can be measured by the ratio of the number of its outgoing edges to the sum of the degrees of its nodes, known as conductance [16]. For example, a cluster has a conductance of 0 if it is disconnected from the rest of the network, and 1 if there are no edges within the cluster.

In order to find clusters in a network, we can use two kinds of algorithms that differ in whether or not they consider the entire graph. A global clustering algorithm seeks to partition the entire graph into clusters, while a local clustering algorithm only considers a small part of the graph close to a given vertex. Much effort has been devoted to using global partitioning algorithms on protein networks [17-22], but the same cannot be said about local techniques. Local algorithms have an obvious advantage because they are often faster. In addition, they may be more useful if the user is only interested in a certain neighborhood of the network. Even though a local algorithm uses less information, it can utilize a local view of the graph to find a quality cluster [23].

### Contribution

The objective of this work is to develop a tool (Local Protein Community Finder) for finding high-quality communities near a queried protein in a PPI network. Here we measure the quality of a community by its conductance, and the functional coherence of its proteins. We expect clusters of interacting proteins to be functional modules, yet this may not always be the case. Therefore, we need to validate the biological significance of the found communities. To evaluate functional coherence, we consider how functionally related the proteins inside the cluster are to each other, and to the other proteins in the network. In order to determine the functional distance between a pair of proteins, we use a measure derived from annotation based on biological literature [24].

The Local Protein Community Finder uses a recently developed local clustering algorithm called Nibble [23], and its extension PageRank-Nibble [25]. Both of them start from a single vertex, and look for a cluster of good conductance in its neighborhood. To our knowledge, our study is the first application of non-heuristic local clustering algorithms to protein networks. We compare Nibble and PageRank-Nibble to Metis, a popular and effective graph partitioning algorithm [26], and a common spectral clustering implementation [27]. Metis partitions the entire network into mutually disjoint balanced clusters, keeping the edge cut, which is the set of edges with endpoints in different clusters, as small as possible. The intuition behind spectral clustering is described in [27-34], and Metis works better than commonly used spectral clustering methods in terms of the size of the resulting edge cut [26]. We show that among the algorithms considered, Nibble and PageRank-Nibble find better clusters in terms of conductance and functional coherence, making our tool useful for finding interesting communities close to a queried protein.

## Methods

### Preliminaries

We model a protein network as an undirected, unweighted graph where the nodes are the proteins, and two nodes are connected by an edge if the corresponding proteins are annotated as interacting with each other.

#### Graph Representation

Formally, a graph is given by a set of vertices *V *and a set of edges *E*. The degree of a node *u *∈ *V*, denoted by *d*(*u*), is the number of edges adjacent to *u*. A graph is often represented by its adjacency matrix. The adjacency matrix of a graph *G *= (*V*, *E*) is defined by

#### Random Walks

We can learn a lot about the structure of a graph by taking a random walk on it. A random walk is a process where at each step we move from some node to one of its neighbors. The transition probabilities are given by edge weights, so in the case of an unweighted network the probability of transitioning from *u *to any adjacent node is 1/*d*(*u*). Thus the transition probability matrix (often called the random walk matrix) is the normalized adjacency matrix where each row sums to one:

Here the *D *matrix is the degree matrix, which is a diagonal matrix given by

In a random walk it is useful to consider a probability distribution vector *p *over all the nodes in the graph. Here *p *is a row vector, where *p*(*u*) is the probability that the walk is at node *u*, and ∑_*u *∈ *V *_*p*(*u*) = 1. Because we transition between nodes with probabilities given by *W*, if *p*_*t *_is the probability distribution vector at time *t*, then *p*_*t*+1 _= *p*_*t*_*W*.

If we modify the random walk to reset at each step with nonzero probability *α*, it will have a unique steady-state probability distribution. This steady-state distribution, which is known as a PageRank vector, is useful because it tells us how much time we will spend at each vertex in a very long random walk on the graph. For starting vector *s*, and reset probability *α*, the PageRank vector pr_*α *_(*s*) is the unique solution of the linear system

The *s *vector specifies the probability distribution for where the walk transitions when it resets. The original PageRank algorithm used a uniform unit vector for *s *[10,11]. PageRank with non-uniform starting vectors is known as personalized PageRank, and has been used in context-sensitive search on the Web [35,36].

#### Partitioning

A common problem in graph theory is to partition the vertices of a graph into clusters while minimizing the number of intercluster edges. A matrix often used for this purpose is the graph Laplacian. The Laplacian matrix of an undirected graph *G *= (*V*, *E*) (with no self-loops) is defined as follows:

In other words, *L*_*G *_= *D*_*G *_- *A*_*G*_. The eigenvectors of *L*_*G *_reveal some structure of the graph [30], and are often used by spectral graph partitioning algorithms.

### Conductance

Conductance measures proportion of outgoing edges of a set of nodes in the graph. Given a graph *G *= (*V*, *E*), and a subset of vertices *S *∈ *V*, let us call the edge boundary of *S *the collection of edges with one point in *S *and the other outside of *S*:

Let us also define the volume of *S *to be the sum of the degrees of its nodes:

The conductance of *S *is then defined as the ratio of the size of its edge boundary to the volume of the smaller side of the partition:

The lower the conductance, the better the cluster. Notice that a cluster can have low conductance without being dense. Using the minimum of vol(*S*) and vol() in the definition disregards vacuous clusters (for example, when *S *= ∅ and  = *V*).

### Nibble

Nibble, the local clustering algorithm of Spielman and Teng [23], works by looking for a cluster of low conductance among the most popular destinations of a short random walk from the starting vertex. The algorithm starts with a probability distribution vector *p *that has all of its probability in the starting vertex, and at every iteration updates *p *by setting *p*_*t *_= *p*_*t*-1_*W*, where *W *is the lazy random walk transition probability matrix. A lazy random walk is a modified random walk where the probability of remaining at each vertex is 1/2; it is used to ensure that the walk reaches a steady state. After each iteration of the random walk, Nibble checks the probability distribution vector for a cluster of low conductance by performing a "sweep" of *p*.

A sweep is a technique for producing a partition (cluster) from a probability distribution vector. The vertices are ordered by degree-normalized probability, and the conductance of the first *j *vertices in the order is computed, where *j *ranges from 1 to the number of non-zero entries in the vector (*N*), returning the set with lowest conductance. More precisely, let *v*_1_,..., *v*_*N *_be an ordering of (nonzero) vertices of *p *such that *p*(*v*_*i*_)/*d*(*v*_*i*_) ≥ *p*(*v*_*i*+1_)/*d*(*v*_*i*+1_). Consider a collection of sweep sets  = {*v*_1_,..., *v*_*j*_}. Let Φ (*p*) be the smallest conductance of any of these sets,

The algorithm finds Φ (*p*) by sorting the entries of *p *by degree normalized probability, and then computing the conductance of each sweep set to find the minimum. The degree of each vertex *v*, denoted by *d*(*v*), is proportional to the amount of probability that *v *has in the stationary distribution of the random walk. Therefore the sweep sets contain vertices that have significantly more probability in *p *than they do in the stationary distribution, meaning that they are visited more often in a walk from the starting node than they are at steady-state.

In order to bound the runtime of the algorithm, Nibble only looks at a small part of the graph close to the starting vertex by using a truncation operation on the probability distribution vector. Given a parameter *ϵ*, after each iteration of the random walk, we set *p*(*v*) = 0 for every *v *such that *p*(*v*) ≤ *ϵ d*(*v*). Nibble takes as input the number of iterations that it performs, as well as *ϵ*, and returns the sweep set of smallest conductance over all iterations.

Deviating from the algorithm presented in [23], we also implement a constrained version of Nibble, which always reports a cluster containing the starting vertex. Here when we perform a sweep of the probability distribution vector, we always put the starting vertex *s *first in the order (set *v*_1 _= *s*), no matter how much probability there is at that vertex. Therefore, Constrained-Nibble only considers sweep sets that include the starting vertex. Similarly, Nibble can also be modified to only consider sweep sets of a certain size, which is useful when we wish to find a cluster in a specified size range.

### PageRank-Nibble

PageRank-Nibble [25] is similar to Nibble in that it looks for a cluster containing the closest vertices to the starting node. However, instead of using an evolving probability distribution of a random walk from starting node *s*, PageRank-Nibble uses a personalized PageRank vector that gives the stationary distribution of a random walk that always returns to *s *when it resets. Once the personalized PageRank vector is computed, the same sweep technique described in the previous section is used to return the cluster of lowest conductance among its sweep sets.

In addition, to bound the amount of time necessary to compute the PageRank vector and perform a sweep, the algorithm uses an approximation of it. The approximation algorithm computes an *ϵ*-approximate PageRank vector by conducting a random walk that only considers vertices *v *that have more than *ϵ d*(*v*) probability in them. The resulting PageRank vector has few non-zero entries, and the amount of error in it for any subset of vertices *S *is less than *ϵ*·vol(*S*).

PageRank-Nibble uses the same sweep technique to find a partition, so it can also be constrained to only consider sweep sets of a certain size, if we wish to find a cluster in a specified size range. Unlike Nibble, PageRank-Nibble always reports a cluster containing the starting vertex because the starting vertex has the most degree normalized probability in the computed PageRank vector. For calculations of PageRank, *α *(the reset probability in the PageRank equation) is typically chosen to be 0.15. However, we find that using a lower value of *α *(such as 0.02) gives us clusters of lower conductance.

### Metis

Metis is a global graph partitioning algorithm that outperforms other state-of-the-art methods [26]. It takes the number of clusters (*k*) as an argument, and maps each vertex to one of *k *balanced clusters, minimizing the size of the edge cut, which is the set of edges with endpoints in different clusters. Metis is a multilevel algorithm, which coarsens the graph to perform the partitioning, and then improves it as the graph is rebuilt. There are two variations of Metis algorithms for partitioning graphs: Kmetis and Pmetis. We try both, and decide to use Pmetis because it gives us clusters of lower conductance. Pmetis works by recursively bisecting the graph, it is slower but returns clusters that are more balanced in size. The partitions reported by Pmetis are almost all of exactly the same size, so to get clusters of a certain size we simply set *k *accordingly. Because Pmetis is a global algorithm, we partition the entire graph once, and for starting vertex *s *return the cluster containing *s*.

### Spectral Clustering

We use a common spectral clustering implementation, taking the first *d *eigenvectors of the Laplacian matrix of the graph (other than the one corresponding to the lowest eigenvalue), to put each vertex in a *d*-dimensional space. We then use *k*-means clustering to partition the vertices into *k *clusters, again choosing *k *to get clusters of the desired size. However, the sizes of the found clusters vary greatly, so we also use a variation where we simply return the *k *closest vertices to the starting vertex in the spectral embedding space. Once again, if we partition the entire graph, we return the cluster that contains the starting vertex.

### Measuring Functional Distance

In order to assess the functional coherence of the found clusters, we use functional distances from Yu et al. [24]. These values are derived using the Gene Ontology (GO) Process classification scheme, where **functional-distance**(*a, b*) is the number of gene pairs that share all of the least common ancestors of *a *and *b *in the classification hierarchy. A low functional distance score means that two proteins are more functionally related, because there are few protein pairs that have the same functional relationship.

The functional distance measure of Yu et al., which the authors refer to as the "total ancestry measure for GO," has the obvious advantage that it considers all known functions of a pair of proteins, allowing for a great degree of precision in assessing functional similarity. Moreover, unlike other methods that derive distances from the GO classification scheme, this method is very resilient to rough functional descriptions, because it still assigns low distances to pairs of proteins that only share very broad terms, as long as there are few other protein pairs that share all of those terms.

Functional distances from [24] can be quite large, yet differences in scores at the low end are more significant than differences at the high end, which is why we take the logarithm in our calculations:

### Calculating Functional Coherence

To determine the functional coherence of community *C *in a protein network represented by a graph *G *= (*V*, *E*), we compute an absolute and a relative functional coherence score. The *absolute functional coherence *of a community is the difference between the average functional distance of two proteins in the network and the average pairwise functional distance of proteins in the community:

The *relative functional coherence *of a community also takes into account how functionally related the proteins inside the community are to the other proteins in the network, and is defined as the difference in average functional distance of intercommunity and intracommunity protein pairs:

### Correlating Conductance and Functional Coherence

To determine whether communities with better conductance are more likely to be functionally coherent, we choose groups of proteins from each network, rank these groups by conductance, absolute, and relative functional coherence, and compute the correlation between the ranks using the Pearson Correlation Coefficient [37]. How to choose the protein groups for this experiment is non-trivial. They cannot be selected by taking random subsets of nodes in each network, which will most certainly produce disconnected groups with bad conductance and functional coherence. Furthermore, they cannot be selected by using algorithms that minimize conductance, which will produce groups with strong bias towards low conductance. A better way to choose these groups is to first randomly select a vertex, and then choose *k *- 1 of its nearest neighbors, breaking ties in distance randomly. Such "random" protein groups will be connected in the network, with reasonable and variable conductance and coherence values. The size of each group is randomly chosen in the range 10 ≤ *k *≤ 40, because we expect biologically relevant communities to be in this size range.

### The Protein Networks

The protein interaction data that we use in our study is from BioGRID [38], Version 2.0.53, updated June 1, 2009. BioGRID lists interacting protein pairs, and for each pair gives the experimental method used to observe the interaction, as well as the source that submitted it. In our study we use several yeast protein-protein interaction (PPI) networks formed from interactions detected by different methods.

Two of the networks, where protein interactions are detected from bait-and-prey type experiments are Affinity Capture-Western (referred to as **ac-western **in the figures), and Affinity Capture-MS (**ac-ms**). These networks tend to be much more cliquish and contain dense components, which is due to the nature of the experiment used to detect the interactions. A single protein (bait) is used to pull in a set of other proteins (prey), and an interaction is predicted either between the bait and each prey (the spoke model), or between every protein in the group (the matrix model) [39]. We also use Two-Hybrid data in our study. Two-Hybrid methods detect binary interactions, therefore PPI networks based on Two-Hybrid data tend to be less dense and cliquish than ones derived from Affinity Capture experiments.

In addition to using a network formed from the union of all Two-Hybrid interactions listed in BioGRID (**two-hybrid**), we also consider a subset of this data submitted by [40] (**two-hybrid-2**). This network is sparser, but is believed to be of higher quality.

## Results and Discussion

**Figure 1:**
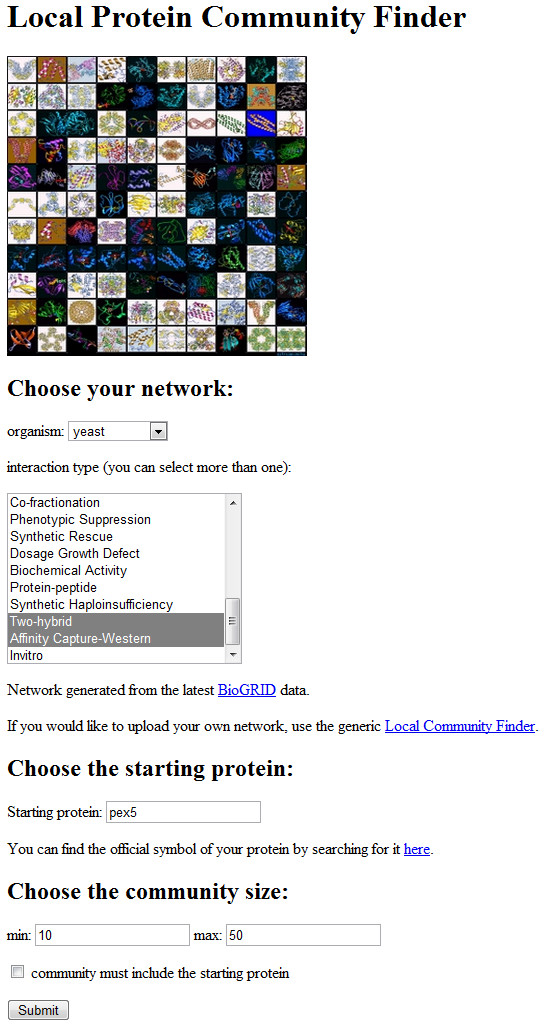
**Local Protein Community Finder user interface**. The user selects a protein network from BioGRID (specified by an organism and a set of interaction types) and a query protein. In addition, the user can choose the size of the community and whether it must contain the starting protein.

**Figure 2:**
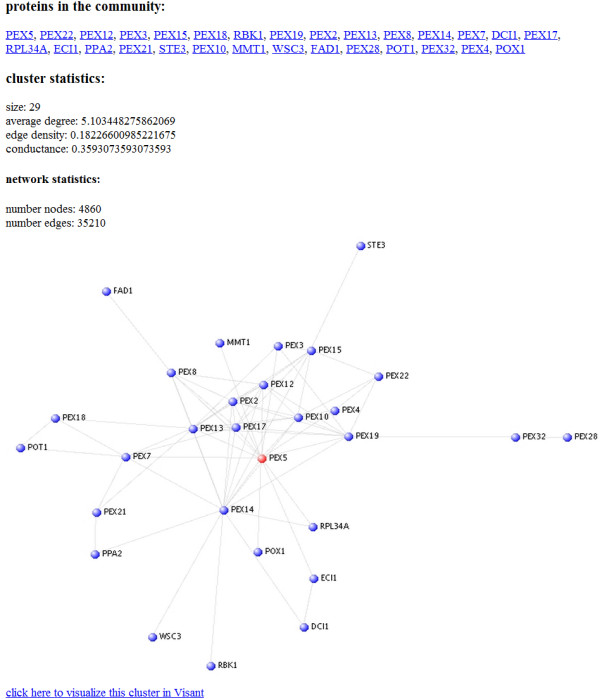
**Local Protein Community Finder output**. An image of the found community is displayed, with the starting protein (if it is part of the community) shown in red, and the other proteins in the community shown in blue. A link to information about each protein is given at the top of the page. The user can also display the community in the protein interaction viewer VisANT by clicking on the link at the bottom of the page.

The Local Protein Community Finder, accessible at http://xialab.bu.edu/resources/lpcf, allows the user to find local communities in any protein network available from BioGRID [38], which is specified by an organism and a set of interaction types. In addition to entering the starting vertex, one can also select the desired cluster size, and whether the reported cluster must contain the starting vertex (Figure 1). Our tool uses the Nibble and PageRank-Nibble algorithms described in Methods, and returns the cluster of lower conductance. The program takes only a few seconds to run, and generates an image of the returned cluster, as well as annotation of the found proteins (Figure 2). In addition, the found community can be displayed in VisANT, a popular protein interaction viewer [41]. If the user would like to use a network that is not from BioGRID, the generic Local Community Finder can be used instead, available at http://xialab.bu.edu/resources/lcf, where one can upload any undirected network in edge-list format. The Local Community Finder also supports weighted networks, as the user can (optionally) specify a weight for each edge. The application is also available as a command-line program (in the form of a single-file Java executable) at http://xialab.bu.edu/resources/lcf/commandLine.

### A Biological Example

First we provide a concrete example where using our tool reveals a biologically meaningful community, while a different commonly used approach does not produce a functionally coherent group. We use our tool to query the local community of yeast protein GET3 in the network from [40], which returns a group of 38 proteins. To investigate the functions of these proteins, we use the GO Term Finder [42] to look for Gene Ontology biological process terms that are significantly enriched in this group (*p*-value ≤ 0.001). GET3 is a subunit of the GET complex, which is involved in Golgi to ER trafficking and insertion of proteins into the ER membrane [43]. We find that the local community of GET3 contains many proteins with similar functions. The community is significantly enriched for relevant Gene Ontology process categories such as protein localization (13 genes, *p *= 2 × 10^-6^), cellular protein localization (12 genes, *p *= 3 × 10^-6^), intracellular protein transport (11 genes, *p *= 9 × 10^-6^), and establishment of localization in cell (15 genes, *p *= 5 × 10^-5^). On the other hand, when we simply take the 38 nearest neighbors of GET3 in the protein network (breaking ties in distance randomly), we get a group of proteins that are not significantly enriched for any of these categories. Thus here it is clear that considering the local community of a protein in a PPI network is much more meaningful than simply looking at its immediate interaction partners.

### Performance Validation

Nibble and PageRank-Nibble have theoretic performance guarantees [23,25], but their effectiveness has not been tested in practice. In order to justify our choice of partitioning algorithms, we compare the performance of Nibble and PageRank-Nibble to two other algorithms commonly used for this purpose: Metis and spectral clustering. We use two variations of spectral clustering: one that uses k-means clustering in the spectral embedding space and reports the cluster containing the starting vertex, and another that simply returns the closest neighbors of the starting vertex in the spectral embedding space (see Methods). To determine whether partitioning a protein interaction network is an effective approach to finding functionally coherent communities, we also compare with a simple nearest neighbor heuristic. Given a starting vertex, nearest neighbor returns the *k *nearest neighbors in the graph, ties broken randomly. Of course, we do not expect this heuristic to return clusters of low conductance, but we do expect it to find functionally coherent groups, because nodes that are connected in a PPI network tend to be much more functionally related [24].

#### Comparing the Algorithms

To compare the performance of the algorithms, we run all of them from the same set of nodes in each PPI network described in Methods, and record the conductance and absolute/relative functional coherence of the found clusters. We then average the statistics of every algorithm in each network, and report the standard error to see if the differences are statistically significant. Moreover, we compare the algorithms when they search for clusters of varying size for the following reasons:

• Functional coherence is sensitive to cluster size (it is usually lower for smaller clusters), therefore we need to control the size of the reported clusters for a rigorous comparison.

• This gives a more thorough performance comparison because it is possible that different algorithms are better at finding clusters of different size.

Thus we compare all methods when they search for small (size 10-20), medium (size 20-30) and large (size 30-40) clusters. We consider this size range because we expect biologically relevant functional groups to be of roughly this size (10-40 proteins). Setting the parameters of each algorithm to only report clusters in a specified size range is straightforward: for *k*-nearest neighbor methods we simply set *k *accordingly; we restrict the local clustering algorithms to only consider sweep sets of a certain size (see Methods); and we vary the desired number of clusters when using the global partitioning algorithms, which gives us clusters in the right size range if the partition is balanced.

#### Comparing Performance in Terms of Conductance

**Figure 3:**
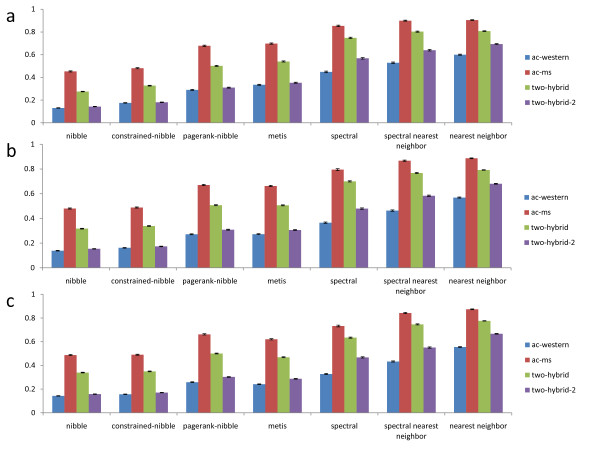
**Average conductance of communities found by each algorithm**. The algorithms compared are listed along the x-axis, the y-axis specifies the average conductance of communities found by each algorithm. Bars of different color are used to represent the results for the four protein networks in which the computational experiments are conducted. (a) The results for small clusters. (b) The results for medium clusters. (c) The results for large clusters.

Figure 3 compares performance of partitioning algorithms in terms of conductance of found clusters. For each network and size range, Nibble finds the lowest conductance clusters, followed by PageRank-Nibble and Metis. Spectral clustering and spectral nearest neighbor do not perform nearly as well. The nearest neighbor heuristic does not take conductance into account, but its results are still reported for completeness. We note that even when we constrain Nibble to only consider clusters that include the starting vertex, it still finds high quality communities. It follows that Nibble does not do better simply because it finds good clusters far from the starting node, while all other algorithms compared here always report a cluster containing the starting vertex.

From Figure 3 we also see that the local clustering algorithms outperform the global ones, which may seem surprising at first. However, a global partitioning algorithm assigns every node to a cluster in order to minimize the edge cut over all clusters in the graph. It is not clear why the same algorithm should be best at minimizing the edge cut for a single cluster in a given neighborhood. The local algorithms, on the other hand, use a view of the graph relative to the starting node, which is specific to each vertex, to find the partition. The intuition for why Nibble works well is that if we take a node inside a cluster of low conductance, and start a random walk from that node, most of the probability will stay inside the cluster because there are proportionally few outgoing edges (and probability in a random walk moves along the edges). Therefore, most of the nodes in the cluster will have a lot of probability in them, and will be in one of the sweep sets (sets of nodes that have the most probability in them) considered by Nibble. Even though PageRank-Nibble does not perform quite as well on all of our data, its theoretic performance guarantee is better than that of Nibble [25], and we believe that it can be especially useful for other networks.

#### Comparing Performance in Terms of Functional Coherence

**Figure 4:**
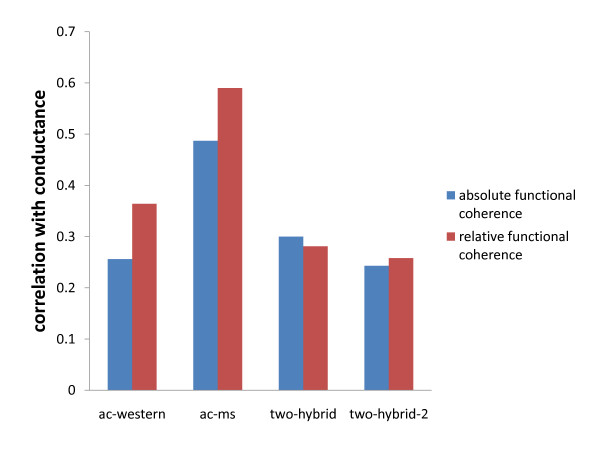
**Correlation between conductance and absolute/relative functional coherence in each network**. The x-axis lists the protein networks. The y-axis gives the Pearson correlation coefficient for the correlation between conductance and functional coherence of protein groups in each network. Correlation between conductance and absolute functional coherence is shown in blue, and correlation between conductance and relative functional coherence is shown in red.

**Figure 5:**
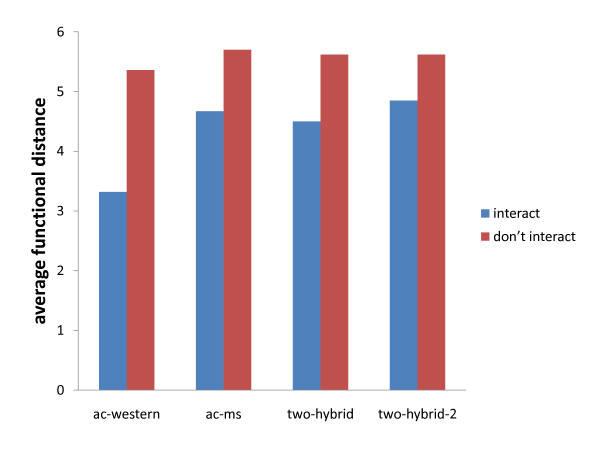
**Average functional distance of interacting and non-interacting protein pairs in each network**. The x-axis lists the protein networks. The y-axis displays the average functional distance of interacting (blue) and non-interacting protein pairs (red) in each network.

In addition to finding good clusters, we would also like our tool to find biologically relevant protein communities. In order to see whether algorithms that minimize conductance are useful for finding functionally coherent groups, we select a large number of protein groups from each network and calculate their conductance and absolute/relative functional coherence, to see if there is any correlation between them (see Methods). The results are presented in Figure 4. We see significant correlation between conductance and both functional coherence measures in all four networks. Moreover, for each network we also compute the average functional distance of interacting and non-interacting proteins (Figure 5). As we expect, in all of our networks interacting proteins are more functionally related. These findings lead us to think that methods that minimize conductance should do well in terms of finding functionally coherent groups in protein networks.

However, we do not expect to see as large a difference in performance between the algorithms used by our tool and other methods in terms of functional coherence of the found communities. There are several reasons for this:

• Our functional coherence calculations depend on the quality of the Gene Ontology annotations from which the pairwise functional distances are derived.

• The protein networks that we use are noisy, and contain false positive and false negative interactions.

• We should not expect methods designed to minimize conductance to perform equally well when their performance is assessed using other, albeit related, measures.

Still, we expect our local partitioning algorithms to find communities with high functional coherence, giving more evidence that the communities reported by our tool are biologically relevant.

**Figure 6:**
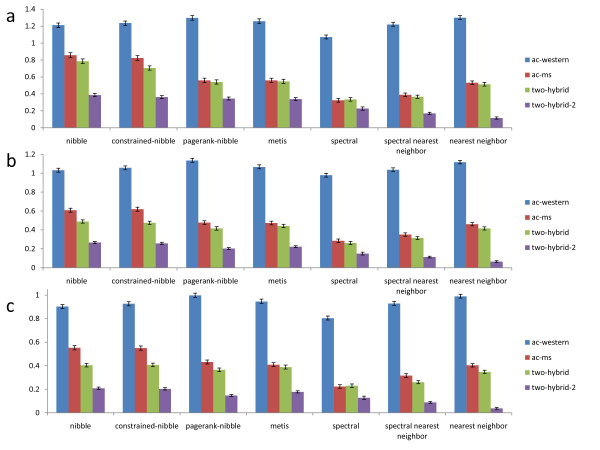
**Average absolute functional coherence of communities found by each algorithm**. The algorithms compared are listed along the x-axis, the y-axis specifies the average absolute functional coherence of communities found by each algorithm. Bars of different color are used to represent the results for the four protein networks in which the computational experiments are conducted. (a) The results for small clusters. (b) The results for medium clusters. (c) The results for large clusters.

Figure 6 compares performance of all methods in terms of absolute functional coherence of found clusters. The coherence of a random subset of proteins in the network is 0, which is also confirmed in our computational experiments. From the figure we can see that in terms of absolute functional coherence, the local partitioning algorithms perform better, or at least as well as other methods. In particular, for small clusters our algorithms significantly outperform other methods in three of the four networks, while for larger clusters the difference in the quality of the communities is smaller. As another negative control, we rewire each network preserving its degree distribution, and record the average coherence of communities found by our algorithms. As expected, the average coherence of these communities is approximately 0 (for all cluster sizes), showing that our results are indeed significant.

**Figure 7:**
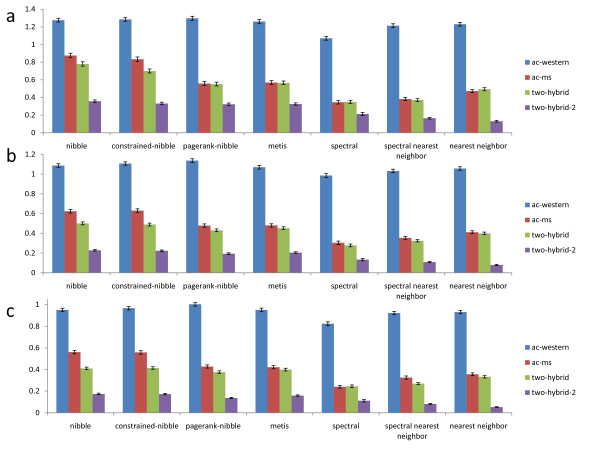
**Average relative functional coherence of communities found by each algorithm**. The algorithms compared are listed along the x-axis, the y-axis specifies the average relative functional coherence of communities found by each algorithm. Bars of different color are used to represent the results for the four protein networks in which the computational experiments are conducted. (a) The results for small clusters. (b) The results for medium clusters. (c) The results for large clusters.

Because our methods minimize conductance, which is a relative measure of edge density, it is more appropriate to evaluate them using a relative measure of functional coherence. Relative functional coherence also takes into account how functionally related the proteins inside the cluster are to the other proteins in the network, and reports the difference in the average functional distance of intercommunity and intracommunity protein pairs. Thus methods that find clusters of low conductance, which are sets of nodes that are better connected among themselves than they are with the rest of the network, should do well in terms of this functional coherence measure. Figure 7 shows that this is indeed the case, as there is a greater contrast in performance between local partitioning algorithms and other methods in terms of relative functional coherence of the found communities. As before, our algorithms significantly outperform other methods when searching for small communities, and the quality of communities found by all algorithms decreases for larger clusters. Once again, the expected coherence of a random subset of proteins in the network is 0, which is also confirmed in our computational experiments. Moreover, when we perform the same rewiring test, the coherence of the found communities is again approximately 0, showing that our results are indeed significant.

## Conclusion

Using conductance to measure the quality of a cluster is especially useful in PPI networks because of the low sampling rate of protein interaction data. The extremely low sampling rate (said to be as low as 3-9% in Two-Hybrid data [39]) presents a problem for any computational method based on PPI network topology. However, undersampling is not likely to affect the conductance of a cluster, because the proportion of outgoing edges is likely to stay the same. Thus if a good cluster exists in the true PPI network, it will also exist in the undersampled network, and will be found by our tool if it is looking in the right neighborhood. On the other hand, algorithms that use absolute criteria in looking for a community (such as seeds of highly-connected nodes, or *k*-cores [44]) will work poorly on PPI data unless these criteria are carefully chosen.

Our results also make an important point about whether it makes sense to use a local partitioning algorithm when it is feasible to use a global one. A local partitioning algorithm only looks at a part of the graph, which gives it an obvious advantage when the graph is very large. However, not much is said about the quality of partitions that a local algorithm finds, compared to a global method. It is easy to think that since a local algorithm only sees a part of the graph, it must always do worse. However, here we show that this is not the case, as the two local partitioning algorithms outperform the global ones. Additionally, the concept of local clustering is quite natural, as often we care about a community local to a part of the graph, rather than how all the nodes cluster. This may be especially true in biology, where researchers typically work on specific proteins of interest.

Finally, we address the biological relevance of communities found in PPI networks. Our results show that by locally partitioning a protein network we can find communities whose proteins are functionally related to each other, and less related to the other proteins in the network.

## Authors' contributions

KV, SHT, and YX conceived and designed the experiments. KV performed the experiments and analyzed the data. KV, SHT, and YX drafted and edited the manuscript.
